# Co-clustering of Time-Dependent Data via the Shape Invariant Model

**DOI:** 10.1007/s00357-021-09402-8

**Published:** 2021-10-06

**Authors:** Alessandro Casa, Charles Bouveyron, Elena Erosheva, Giovanna Menardi

**Affiliations:** 1 School of Mathematics & Statistics, Vistamilk SFI Research Centre, University College Dublin, Belfield, Dublin 4, Ireland; 2 INRIA, CNRS, Laboratoire J.A. Dieudonné, MAASAI research team, Université Côte d’Azur, Nice, France; 3 Department of Statistics, University of Washington, Seattle, WA USA; 4 Deparment of Statistical Sciences, University of Padova, Padua, Italy

**Keywords:** Co-clustering, Curve registration, Latent block model, Stochastic EM

## Abstract

Multivariate time-dependent data, where multiple features are observed over time for a set of individuals, are increasingly widespread in many application domains. To model these data, we need to account for relations among both time instants and variables and, at the same time, for subject heterogeneity. We propose a new co-clustering methodology for grouping individuals and variables simultaneously, designed to handle both functional and longitudinal data. Our approach borrows some concepts from the *curve registration* framework by embedding the *shape invariant model* in the *latent block model*, estimated via a suitable modification of the SEM-Gibbs algorithm. The resulting procedure allows for several user-defined specifications of the notion of cluster that can be chosen on substantive grounds and provides parsimonious summaries of complex time-dependent data by partitioning data matrices into homogeneous blocks. Along with the explicit modelling of time evolution, these aspects allow for an easy interpretation of the clusters, from which also low-dimensional settings may benefit.

## Introduction

Time-dependent data, arising when measurements are taken on a set of units at different time occasions, are pervasive in a plethora of different fields. Examples include, but are not limited to, time evolution of asset prices and volatility in finance, growth of countries as measured by economic indices, heart or brain activities as monitored by medical instruments, disease evolution evaluated by suitable bio-markers in epidemiology. In this heterogeneous landscape, we may distinguish, from a modelling perspective, between functional and longitudinal settings. In the former case, a large number of regularly sampled observations are usually available, allowing to treat each element of the sample as a function. In longitudinal studies, conversely, only a few observations over time are typically available with sparse and irregular measurements. Readers may refer to Rice (2004) for a thorough comparison and discussion about differences and similarities between functional and longitudinal data analysis.

Early developments in these areas mainly aim to describe individual-specific curves by properly accounting for the correlation between measurements for each subject (see, e.g. Diggle et al., 2002; Ramsay and Silverman, 2005and references therein) with the subjects themselves often considered to be independent. This is not always the case; hence, more recently, there has been increasing interest in clustering methodologies aimed at describing heterogeneity among time-dependent observed trajectories; see Erosheva et al. (2014) for a recent review of related methods used in criminology and developmental psychology. From a functional standpoint, different approaches have been studied and readers may refer to the works by Bouveyron and Jacques(2011), Bouveyron et al. (2015) and Bouveyron et al. (2020) or to Jacques & Preda (2014) for a review. On the other hand, from a longitudinal point of view, De la Cruz-Mesía et al. (2008), McNicholas & Murphy (2010) proposed model-based clustering approaches. Lastly, a review from a more general time-series perspective may be found in Liao (2005) and Frühwirth-Schnatter (2011).

**Fig. 1:**
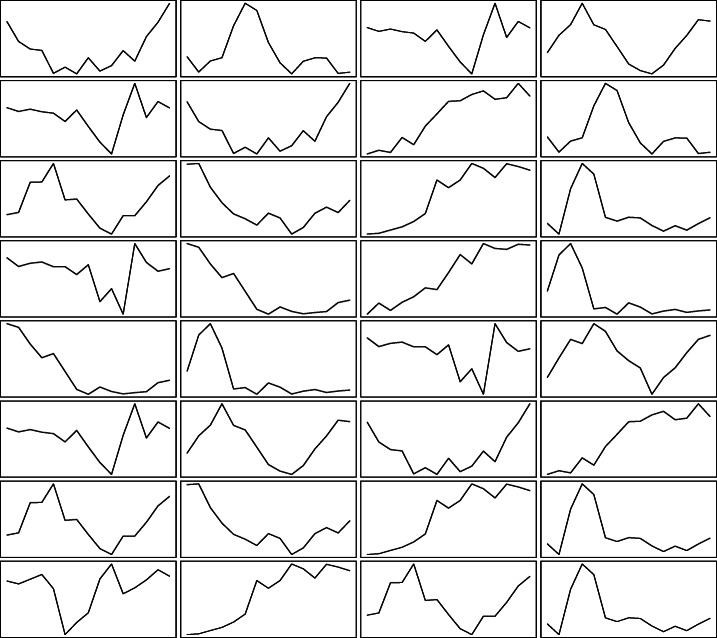
Example of multivariate time-dependent data: *d* = 4 variables are measured for *n* = 8 individuals over *T* = 15 time instants, giving rise to the displayed curves

The methods cited so far usually deal with situations where a single feature is measured over time for a number of subjects, where the data are represented by a *n* × *T* matrix, with *n* being the number of subjects and *T* being the number of observed time occasions. In fact, it is increasingly common to encounter multivariate time-dependent data, with several variables measured over time for different individuals. These data may be represented according to three-way *n* × *d* × *T* matrices, with *d* being the number of time-dependent features; a graphical illustration of such structure is displayed in Fig. 1. The introduction of an additional layer entails new challenges that have to be faced by clustering tools. As noted by (Anderlucci and Viroli, 2015), models have to account for three different aspects, being the correlation across different time observations, the relationships between the variables and the heterogeneity among the units, each one of them arising from a different layer of the three-way data structure.

To extract useful information and to unveil patterns from such complex structured and high-dimensional data, standard clustering strategies would require specification and estimation of highly parameterized models. In this situation, parsimony is often induced by neglecting the correlation structure among variables. An alternative approach, specifically proposed in a parametric setting, is represented by the contributions of Viroli (2011a, ??) which exploit mixtures of Gaussian matrix-variate distributions, in order to handle three-way data.

In the present work, we take a different direction, by pursuing a co-clustering strategy to address the mentioned issues. The term co-clustering refers to those methods finding row and column clusters of a data matrix simultaneously. These techniques are particularly useful in high-dimensional settings where standard clustering methods may fall short in uncovering meaningful and interpretable row groups because of the high number of variables. By searching for homogeneous blocks in large matrices, co-clustering tools produce parsimonious summaries that could provide useful lower dimensional representations of the data. These techniques are particularly appropriate when relations among the observed variables are of interest. Note that, even in the co-clustering context, the usual dualism between *distance-based* and *density-based* strategies can be found. We pursue the latter approach, which embeds co-clustering in a probabilistic framework, builds a common setting to handle different types of data, and reflects the idea of a density resulting from a mixture model. Specifically, we propose a parametric model for time-dependent data and a new estimation strategy to handle the distinctive characteristics of the model. Parametric co-clustering of time-dependent data has been pursued by Ben Slimen et al. (2018) and Bouveyron et al. (2018) in a functional setting, by mapping the original curves to the space spanned by the coefficients of a basis expansion. By modelling explicitly the observed data, instead of basis expansion coefficients, we provide a natural description of the time evolution and facilitate cluster interpretation. The proposed model builds on the idea that individual curves within a cluster arise as transformations of a common shape function, which is in turn modeled to handle both functional and longitudinal data, regardless of their dimensionality. Lastly, the framework we develop allows for a flexible specification of different notions of clusters, possibly depending on subject matter considerations.

The rest of the paper is organized as follows. In Section 2, we provide the background needed for the specification of the proposed model which is described in Section 3, along with the estimation procedure. In Section 4, the model performances are illustrated both on simulated and real examples. In Section 5, we conclude the paper by summarizing our contributions and pointing to some future research directions.

## Modelling Time-Dependent Data

In the heterogeneous time-dependent data landscape outlined in the previous section, it is sensible to pursue a variety of modelling approaches. The route we follow borrows its rationale from the *curve registration* framework (Ramsay and Li, 1998), according to which observed curves often exhibit common patterns but with some variations. Methods for curve registration, also known as *curve alignment* or *time warping*, are based on the idea of aligning prominent features in a set of curves via either an *amplitude variation*, a *phase variation* or a combination of the two. The first one concerns vertical variations while the latter regards horizontal, hence time related, ones. As an example, it is possible to think about modelling the evolution of a specific disease. Here, the observable heterogeneity of the raw curves can often be disentangled in two distinct sources: on the one hand, it could depend on differences in the intensities of the disease among subjects whereas, on the other hand, there could be different ages of onset, i.e. the age at which an individual experiences the first symptoms. After properly taking into account these causes of variation, the curves result to be more homogeneously behaving, with a so-called *warping function*, which synchronizes the observed curves and allows for visualization and estimation of a common mean shape curve.

Similarly, in this work, we account for time-dependency via a *self-modelling regression* approach (Lawton et al., 1972) and, more specifically, via an extension of the so-called *shape invariant model* (SIM, Lindstrom, 1995), based on the idea that an individual curve arises as a simple transformation of a common shape function.

Let $\mathcal {X}=\{x_{i}(\mathbf {t}_{i})\}_{1\le i \le n}$ be the set of curves, observed on *n* individuals, with *x*_*i*_(*t*) being the level of the *i* th curve at time *t* and $t \in \mathbf {t}_{i} = (t_{i,1}, \dots , T_{i,n_{i}})$, hence with the time points and their number allowed to be subject-specific. Stemming from the SIM, *x*_*i*_(*t*) is modelled as

1 $$ \begin{array}{@{}rcl@{}} x_{i}(t) = \alpha_{i,1} + \text{e}^{\alpha_{i,2}}m(t-\alpha_{i,3}) + \epsilon_{i}(t)  , \end{array} $$

where

- *m*(⋅) denotes a general common shape function whose specification is arbitrary. In the following we consider B-spline basis functions (De Boor, 1978), i.e. letting $m(t)=m(t;\beta )= {\mathscr{B}}(t)\beta ,$ where ${\mathscr{B}}(t)$ and *β* are respectively a vector of B-spline basis functions evaluated at time *t* and a vector of basis coefficients whose dimensions allow for different degrees of flexibility;
- $\alpha _{i}=(\alpha _{i,1},\alpha _{i,2},\alpha _{i,3}) \sim \mathcal {N}_{3}(\mu ^{\alpha },{\Sigma }^{\alpha })$ for $i=1,\dots ,n$ is a vector of subject-specific normally distributed random effects. These random effects are responsible for the individual specific transformations of the mean shape curve *m*(⋅) assumed to generate the observed ones. In particular, *α*_*i*,1_ and *α*_*i*,3_ govern, respectively, amplitude and phase variations while *α*_*i*,2_ describes possible scale transformations. Random effects also account for the correlation among observations on the same subject measured at different time points;
- $\epsilon _{i}(t) \sim \mathcal {N}(0,\sigma ^{2}_{\epsilon })$ is a Gaussian distributed error term.

Due to its flexibility, Telesca & Inoue (2008) and Telesca et al. (2012) have already considered the SIM as a stepping stone to model both functional and longitudinal time-dependent data. Indeed, on the one hand, the smoothing involved in the specification of *m*(⋅;*β*) allows to handle function-like data. On the other hand, random effects, which borrow information across curves, make this approach fruitful even with short, irregular and sparsely sampled time series; readers may refer to Erosheva et al. (2014) for an illustration in the context of behavioral trajectories. Therefore, we find model in Eq. 1 particularly suitable for our aims, potentially being able to handle time-dependent data in a comprehensive way.

## Time-Dependent Latent Block Model

### Latent Block Model

In the parametric, or model-based, co-clustering framework, the *latent block model* (LBM, Govaert and Nadif, 2013) is the most popular approach. Data are represented in a matrix form $\mathcal {X}=\{ x_{ij} \}_{1\le i \le n, 1 \le j \le d}$, where by now we should intend *x*_*ij*_ as a generic random variable. To aid the definition of the model, and in accordance with the parametric approach to clustering (Fraley & Raftery, 2002; Bouveyron et al., 2019), two latent random vectors **z** = {*z*_*i*_}_1≤*i*≤*n*_, and **w** = {*w*_*j*_}_1≤*j*≤*d*_, with $z_{i} = (z_{i1},\dots ,z_{iK})$, $w_{j}=(w_{j1},\dots ,w_{jL})$, are introduced, indicating respectively the row and column memberships, with *K* and *L* the number of row and column clusters. A standard binary notation is used for the latent variables, i.e. *z*_*ik*_ = 1 if the *i* th observation belongs to the *k* th row cluster and 0 otherwise and, likewise, *w*_*jl*_ = 1 if the *j* th variable belongs to the *l* th column cluster and 0 otherwise. The model formulation relies on a local independence assumption, where the *n* × *d* random variables {*x*_*ij*_}_1≤*i*≤*n*,1≤*j*≤*d*_, are assumed to be independent conditionally on **z** and **w**, in turn supposed to be independent. The LBM can be thus written as

2 $$ \begin{array}{@{}rcl@{}} p(\mathcal{X}; {\Theta}) = {\sum}_{z \in Z}{\sum}_{w \in W}p(\mathbf{z};{\Theta})p(\mathbf{w};{\Theta})p(\mathcal{X}|\mathbf{z},\mathbf{w};{\Theta})  , \end{array} $$

where

- *Z* and *W* are the sets of all the possible partitions of rows and columns respectively in *K* and *L* groups;
- the latent vectors **z**,**w** follow a multinomial distribution, with $p(\mathbf {z};{\Theta })={\prod }_{ik}\pi _{k}^{z_{ik}},$ $p(\mathbf {w};{\Theta })={\prod }_{jl} \rho _{l}^{w_{jl}}$ and *π*_*k*_,*ρ*_*l*_ > 0 are the row and column mixture proportions, ${\sum }_{k} \pi _{k} = {\sum }_{l} \rho _{l} = 1$;
- as a consequence of the local independence assumption, $p(\mathcal {X}|\mathbf {z},\mathbf {w};{\Theta }) = {\prod }_{ijkl} p(x_{ij};\theta _{kl})^{z_{ik}w_{jl}}$ where *𝜃*_*kl*_ is the vector of parameters specific to block (*k*,*l*);
- Θ = (*π*_*k*_,*ρ*_*l*_,*𝜃*_*kl*_)_1≤*k*≤*K*,1≤*l*≤*L*_ is the full parameter vector of the model.

The LBM is particularly flexible in modelling different data types, as handled by a proper specification of the marginal density *p*(*x*_*ij*_;*𝜃*_*kl*_) for binary (Govaert & Nadif, 2003), count (Govaert & Nadif, 2010), continuous (Lomet, 2012), categorical (Keribin et al., 2015), ordinal (Jacques & Biernacki, 2018; Corneli et al., 2020) and even mixed-type data (Selosse et al., 2020).

### Model Specification

Once the LBM structure has been properly defined, extending its rationale to handle time-dependent data in a co-clustering framework boils down to a suitable specification of *p*(*x*_*ij*_;*𝜃*_*kl*_). Note that this reveals one of the main advantages of such a highly-structured model, consisting in the chance to search for patterns in multivariate and complex data by specifying only the model for the variable *x*_*ij*_. As introduced in Section 1, multidimensional time-dependent data may be represented according to a three-way structure where the third *mode* accounts for the time evolution. The observed data assume an array configuration $\mathcal {X}= \{ x_{ij}(\mathbf {t}_{i}) \}_{1\le i \le n, 1\le j \le d}$ with $\mathbf {t}_{i}=(t_{i,1},\dots ,T_{i,n_{i}})$ as outlined in Section 2; from a practical standpoint, subject dependent time instants, sparsely sampled curves and different observational lengths can be handled by a suitable use of missing entries. Consistently with Eq. 1, we consider as a generative model for the curve in the (*i*,*j*)th entry, belonging to the generic block (*k*,*l*), the following

3 $$ \begin{array}{@{}rcl@{}} x_{ij}(t)|_{z_{ik}=1,w_{jl}=1} = \alpha_{ij,1}^{kl} + \text{e}^{\alpha_{ij,2}^{kl}}m(t-{\alpha}_{ij,3}^{kl}; \beta_{kl}) + \epsilon_{ij}(t)  , \end{array} $$

with *t* ∈**t**_*i*_ a generic time instant. A relevant difference with respect to the original SIM consists, coherently with the co-clustering setting, in the parameters being block-specific since the generative model is specified conditionally to the block membership of the cell. As a consequence:

- $m(t;\beta _{kl})= {\mathscr{B}}(t)\beta _{kl}$ where the quantities are defined as in Section 2, with the only difference that *β*_*kl*_ is a vector of block-specific basis coefficients, hence allowing different mean shape curves across different blocks;
- $\alpha _{ij}^{kl}=({\alpha }_{ij,1}^{kl},{\alpha }_{ij,2}^{kl},{\alpha }_{ij,3}^{kl}) \sim \mathcal {N}_{3}(\mu _{kl}^{\alpha },{\Sigma }_{kl}^{\alpha })$ is a vector of cell-specific random effects distributed according to a block-specific Gaussian distribution;
- $\epsilon _{ij}(t) \sim \mathcal {N}(0,\sigma ^{2}_{\epsilon ,kl})$ is the error term distributed as a block-specific Gaussian;
- $\theta _{kl}=(\mu _{kl}^{\alpha },{\Sigma }_{kl}^{\alpha },\sigma ^{2}_{\epsilon ,kl},\beta _{kl})$.

**Fig. 2:**
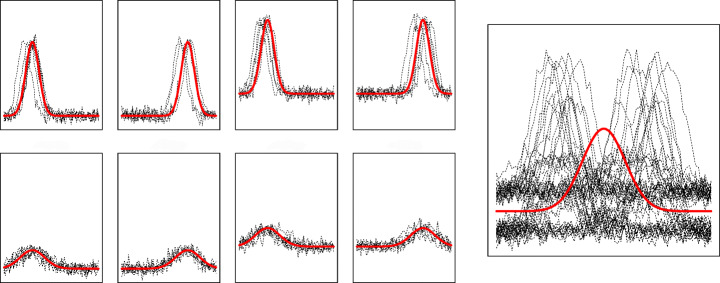
In the left panels, curves in dotted line arise as random fluctuations of the superimposed red curve, but they are all time, amplitude or scale transformations of the same mean-shape function on the right panel

Note that here we embed the ideas borrowed from the *curve registration* framework in a clustering setting. Therefore, while *curve alignment* aims to synchronize the curves to estimate a common mean shape, in our setting the SIM works as a suitable tool to model the heterogeneity inside a block and to introduce a flexible notion of cluster. The rationale behind considering the SIM in a co-clustering framework consists in looking for blocks characterized by a different mean shape function *m*(⋅;*β*_*kl*_). Curves within the same block arise as random shifts and scale transformations of *m*(⋅;*β*_*kl*_), driven by the block-specifically distributed random effects. Let consider the small panels on the left side of Fig. 2, displaying a number of curves which arise as transformations induced by non-zero values of *α*_*ij*,1_, *α*_*ij*,2_, or *α*_*ij*,3_. Beyond the sample variability, the curves differ for a (phase) random shift on the *x* −axes, an amplitude shift on the *y*-axes, and a scale factor. According to model Eq. 3, all those curves belong to the same cluster, since they share the same mean shape function (Fig. 2, right panel).

Further flexibility can be naturally introduced within the model by “switching off” one or more random effects depending on subject-matter considerations and on the user’s cluster definition. For example, if there are reasons to support that similar, yet shifted in time, evolutions are expressions of different clusters, it makes sense to switch off *α*_*ij*,3_. As a consequence, the model specification in Eq. 3 would no longer include the corresponding random effect *α*_*ij*,3_

$$ \begin{array}{@{}rcl@{}} x_{ij}(t)|_{z_{ik}=1,w_{jl}=1} = \alpha_{ij,1}^{kl} + \text{e}^{\alpha_{ij,2}^{kl}}m(t; \beta_{kl}) + \epsilon_{ij}(t)  . \end{array} $$

**Fig. 3:**
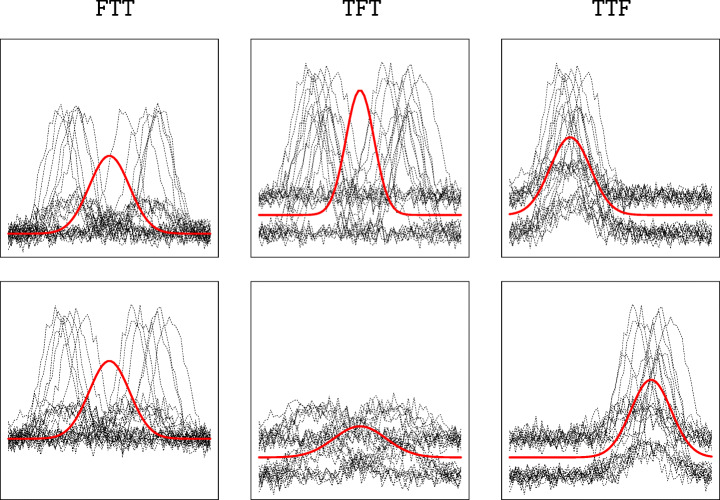
Pairs of plots in each column represent the two-cluster configurations arising from switching off, from left to right *α*_*ij*,1_, *α*_*ij*,2_, *α*_*ij*,3_. In the names of the models, as used in the rest of the paper, T indicates a switched on random effect while F a switched off one

In the following, we refer to this model as TTF, to highlight that the third random effect is switched off. In the example illustrated in Fig. 2 this situation ideally leads to a two-cluster structure (Fig. 3, right panels). Similarly, if comparable time evolution curves associated to different intensities are seen as expressions of distinct groups, the random intercept *α*_*ij*,1_ can be switched off, and we refer to this class of models as FTT. Lastly, removing *α*_*ij*,2_ results in TFT models which would determine different blocks varying for a scale factor (Fig. 3, middle panels). From a practical standpoint, switching off a random effect amounts to constrain it to follow a degenerate distribution centered at zero in the estimation scheme outlined in the next section.

### Model Estimation

To estimate the LBM several approaches have been proposed, as for example Bayesian estimation (Wyse & Friel, 2012), greedy search algorithms (Wyse et al., 2017) and likelihood-based procedures (Govaert & models, 2008). In this work we focus on the latter class of methods. In principle, the estimation strategy would aim to maximize the log-likelihood $\ell ({\Theta }) = \log p(\mathcal {X}; {\Theta })$ with $p(\mathcal {X}; \theta )$ defined as in Eq. 2; nonetheless, the missing structure of the data makes this maximization impractical. For this reason the *complete-data log-likelihood* is usually considered as the objective function to optimize, defined as

4 $$  \ell_{c}({\Theta},\mathbf{z},\mathbf{w}) = \sum\limits_{ik} z_{ik}\log\pi_{k} + \sum\limits_{jl}w_{jl}\log\rho_{l} + \sum\limits_{ijkl}z_{ik}w_{jl}\log p(x_{ij}; \theta_{kl})  , $$

where the first two terms account for the proportions of row and column clusters while the third one depends on the probability density function of each block.

As a general solution, to maximize Eq. 4 and obtain an estimate of $\hat {\Theta }$ when dealing with situations where latent variables are involved, one would in principle resort to the expectation-maximization algorithm (EM, Dempster et al., 1977). The basic idea underlying the EM algorithm consists in finding a lower bound of the log-likelihood and optimizing it via an iterative scheme in order to create a converging series of $\hat {\Theta }^{(h)}$. In the co-clustering framework, this lower bound can be easily exhibited by rewriting the log-likelihood as follows

$$ \ell({\Theta}) = \mathcal{L}(q;{\Theta}) + \zeta  , $$

where ${\mathscr{L}}(q; {\Theta }) = {\sum }_{\mathbf {z},\mathbf {w}} q(\mathbf {z},\mathbf {w})\log (p(\mathcal {X},\mathbf {z},\mathbf {w}| \theta )/q(\mathbf {z},\mathbf {w})),$ *q*(**z**,**w**) is a generic probability mass function on the support of (**z**,**w**) while *ζ* is a positive constant not depending on Θ.

The E step of the algorithm maximizes the lower bound ${\mathscr{L}}$ over *q* for a given value of Θ. Straightforward calculations show that ${\mathscr{L}}$ is maximized for $q^{*}(\mathbf {z},\mathbf {w})=p(\mathbf {z},\mathbf {w}|\mathcal {X},\theta )$. Unfortunately, in a co-clustering scenario, the joint posterior distribution $p(\mathbf {z},\mathbf {w}|\mathcal {X},{\Theta })$ is not tractable, as it involves terms that cannot be factorized as it conversely happens in a standard mixture model framework. As a consequence, several modifications have been explored, searching for viable solutions when performing the E step (see Govaert & Nadif, 2013 for a more detailed tractation); examples are the *classification EM* (CEM) and the *variational EM* (VEM). Here we propose to make use of a Gibbs sampler within the E step to approximate the posterior distribution $p(\mathbf {z},\mathbf {w}|\mathcal {X},{\Theta })$. This results in a stochastic version of the EM algorithm, which will be called SEM-Gibbs in the following. Given an initial column partition **w**^(0)^ and an initial value for the parameters Θ^(0)^, at the *h* th iteration the algorithm proceeds as follows:

- SE step: $q^{*}(\mathbf {z},\mathbf {w})\simeq p(\mathbf {z},\mathbf {w}|\mathcal {X},{\Theta }^{(h-1)})$ is approximated with a Gibbs sampler. The Gibbs sampler consists in sampling alternatively **z** and **w** from their conditional distributions a certain number of times before to retain new values for **z**^(*h*)^ and **w**^(*h*)^,
- M step: ${\mathscr{L}}(q^{*}(\mathbf {z}^{(h)},\mathbf {w}^{(h)}),{\Theta }^{(h-1)})$ is then maximized over Θ, where
  $$ \begin{array}{@{}rcl@{}} \mathcal{L}(q^{*}(\mathbf{z}^{(h)},\mathbf{w}^{(h)}),{\Theta}^{(h-1)}) & \simeq & \sum\limits_{z,w}p(\mathbf{z},\mathbf{w}|\mathcal{X},{\Theta}^{(h-1)})\log(p(\mathcal{X},\mathbf{z},\mathbf{w}|{\Theta})/p(\mathbf{z},\mathbf{w}|\mathcal{X},{\Theta}^{(h-1)}))\\ & \simeq & E[\ell_{c}({\Theta}, \mathbf{z}^{(h)}, \mathbf{w}^{(h)})|{\Theta}^{(h-1)}]+\xi  , \end{array} $$
  *ξ* not depending on Θ. This step therefore reduces to the maximization of the conditional expectation of the *complete-data log-likelihood* in Eq. 4 given **z**^(*h*)^ and **w**^(*h*)^.

In the proposed framework, due to the presence of the random effects, some additional challenges have to be faced. In fact, the maximization of the conditional expectation of Eq. 4 associated to model in Eq. 3 requires a cumbersome multidimensional integration in order to compute the marginal density defined as

5 $$ \begin{array}{@{}rcl@{}} p(x_{ij};\theta_{kl}) = \int p(x_{ij}|\alpha_{ij}^{kl};\theta_{kl})p(\alpha_{ij}^{kl};\theta_{kl}) d\alpha_{ij}^{kl}  . \end{array} $$

Note that, with a slight abuse of notation, we suppress the dependency on the time *t*, i.e. *x*_*ij*_ will represent *x*_*ij*_(**t**_*i*_). In the SE step, on the other hand, the evaluation of Eq. 5 is needed for all the possible configurations of $\{z_{i}\}_{i=1,\dots ,n}$ and $\{w_{j}\}_{j=1,\dots ,d}$. These quantities are obtained when the SEM-Gibbs is used to estimate models without any random effect involved, while their computation is more troublesome in our scenario.

We propose a modification of the SEM-Gibbs algorithm, called *marginalized SEM-Gibbs* (M-SEM), where an additional *marginalization step* is introduced to account for the random effects. Given an initial value for the parameters Θ^(0)^ and an initial column partition **w**^(0)^, the *h*-th iteration of the M-SEM algorithm alternates the following steps:

- **Marginalization step**: The single cell contributions in Eq. 5 to the *complete-data log-likelihood* are computed by means of a Monte Carlo integration scheme as
  6 $$ \begin{array}{@{}rcl@{}} p(x_{ij};\theta_{kl}^{(h-1)}) \simeq \frac{1}{M} \sum\limits_{m=1}^{M} p(x_{ij} ; \alpha_{ij}^{kl,(m)}, \theta_{kl}^{(h-1)})  , \end{array} $$
  for $i=1,\dots ,n$, $j=1,\dots ,d$, $k=1,\dots ,K$ and $l=1,\dots ,L$ and *M* being the number of Monte Carlo samples. The values of the vectors $\alpha _{ij}^{kl,(1)},\dots ,\alpha _{ij}^{kl,(M)}$ are drawn from a Gaussian distribution $\mathcal {N}_{3}(\mu _{kl}^{\alpha ,(h-1)},{\Sigma }_{kl}^{\alpha ,(h-1)})$ with this choice amounting to a random version of the *Gaussian quadrature rule* (Pinheiro & Bates, 2006). Whenever one or more random effects are not included in the model (i.e. they are switched off), the corresponding draws come from degenerate random variables, and set to zero in the estimation process.
- **SE step**: $p(\mathbf {z},\mathbf {w}|\mathcal {X},{\Theta }^{(h-1)})$ is approximated by repeating, for a number of iterations, the following Gibbs sampling steps
  1. generate the row partition $z_{i}^{(h)}=(z_{i1}^{(h)},\dots ,z_{iK}^{(h)}), i=1,\dots ,n$ according to a multinomial distribution $z_{i}^{(h)}\sim {\mathscr{M}}(1,\tilde {z}_{i1},\dots ,\tilde {z}_{iK})$, with
    $$ \begin{array}{@{}rcl@{}} \tilde{z}_{ik} &=& p(z_{ik}=1 | \mathcal{X},\mathbf{w}^{(h-1)};{\Theta}^{(h-1)}) \\ &=& \frac{\pi_{k}^{(h-1)}p_{k}(\mathbf{x}_{i} | \mathbf{w}^{(h-1)}; {\Theta}^{(h-1)})}{{\sum}_{k^{\prime}}\pi_{k^{\prime}}^{(h-1)}p_{k^{\prime}}(\mathbf{x}_{i} | \mathbf{w}^{(h-1)}; {\Theta}^{(h-1)})}  , \end{array} $$
    for $k=1,\dots ,K$, with **x**_*i*_ = {*x*_*ij*_}_1≤*j*≤*d*_ the *i* th row of $\mathcal {X}$ and $p_{k}(\mathbf {x}_{i} | \mathbf {w}^{(h-1)}; {\Theta }^{(h-1)}) = {\prod }_{jl} p(x_{ij}; \theta _{kl}^{(h-1)})^{w_{jl}^{(h-1)}}$.
  2. generate the column partition $w_{j}^{(h)}=(w_{j1}^{(h)},\dots ,w_{jL}^{(h)}), j=1,\dots ,d$ according to a multinomial distribution $w_{j}^{(h)}\sim {\mathscr{M}}(1,\tilde {w}_{j1},\dots ,\tilde {w}_{jL})$, with
    $$ \begin{array}{@{}rcl@{}} \tilde{w}_{jl} &=& p(w_{jl}=1 | \mathcal{X}, \mathbf{z}^{(h)}; {\Theta}^{(h-1)}) \\ &=& \frac{\rho_{l}^{(h-1)}p_{l}(\mathbf{x}_{j} | \mathbf{z}^{(h)}; {\Theta}^{(h-1)})}{{\sum}_{l^{\prime}}\rho_{l^{\prime}}^{(h-1)}p_{l^{\prime}}(\mathbf{x}_{j} | \mathbf{z}^{(h)}; {\Theta}^{(h-1)})}  , \end{array} $$
    for $l=1,\dots ,L$, with **x**_*j*_ = {*x*_*ij*_}_1≤*i*≤*n*_ the *j* th column of $\mathcal {X}$ and $p_{l}(\mathbf {x}_{j} | \mathbf {z}^{(h)}; {\Theta }^{(h-1)}) = {\prod }_{ik} p(x_{ij}; \theta _{kl}^{(h-1)})^{z_{ik}^{(h)}}$.
- **M step**: Estimate Θ^(*h*)^ by maximizing $E[\ell _{c}({\Theta }, \mathbf {z}^{(h)}, \mathbf {w}^{(h)})|{\Theta }^{(h-1)}]$. Update mixture proportions as $\pi _{k}^{(h)} = \frac {1}{n}{\sum }_{i}z_{ik}^{(h)}$ and $\rho _{l}^{(h)}=\frac {1}{d}{\sum }_{j} w_{jl}^{(h)}$. The estimate of $\theta _{kl}=(\mu _{kl}^{\alpha },{\Sigma }_{kl}^{\alpha },\sigma ^{2}_{\epsilon ,kl},\beta _{kl})$ is obtained by exploiting the *non-linear mixed effect model* specification in Eq. 3 and considering the approximate maximum likelihood formulation proposed in Lindstrom and Bates (1990); the variance and the mean components are estimated by approximating and maximizing the marginal density of the latter near the mode of the posterior distribution of the random effects. Conditional or shrinkage estimates are then used for the estimation of the random effects.

The M-SEM algorithm is run until a convergence criterion is met. The convergence for the proposed procedure is assessed by monitoring the evolution of the *complete-data log-likelihood*: more specifically the algorithm reaches convergence when the sum of the changes in *ℓ*_*c*_(Θ,**z**,**w**) in the last three iterations are smaller than a given threshold *δ* > 0. Since a burn-in period is considered, the final estimate for Θ, denoted as $\hat {\Theta }$, is given by the mean of the sample distribution. A sample of (**z**,**w**) is then generated according to the SE step as illustrated above with ${\Theta }=\hat {\Theta }$. The final block-partition $(\hat {\mathbf {z}},\hat {\mathbf {w}})$ is then obtained as the mode of their sample distribution.

The approach considered in this work represents an extension to the likelihood maximization strategies, usually adopted in the LBM framework. Note that other choices could be alternatively explored, such as fully Bayesian estimation schemes that would allow for statistical inference on the parameter estimates (van Dijk et al., 2009) and for the automatic selection of the number of blocks (Wyse & Friel, 2012).

### Model Selection

The choice of the number of groups is considered here as a model selection problem. Operationally we estimate several models, corresponding to different combinations of *K* and *L* and, in our case, to different configurations of the random effects, and we select the best one according to an information criterion. Note that the model selection step is more troublesome in this setting with respect to a standard clustering one, since we need to select not only the number of row clusters *K* but also the number of column ones *L*. Standard choices, such as the AIC and the BIC, are not directly available in the co-clustering framework where, as noted by Keribin et al. (2015), the computation of the likelihood of the LBM is challenging, even when the parameters are properly estimated. A viable alternative is to consider an approximated version of the ICL (Biernacki et al., 2000) that, relying on the *complete-data log-likelihood*, does not suffer from the same issues:

7 $$ \begin{array}{@{}rcl@{}} \text{ICL} = \ell_{c}(\hat{\Theta}, \hat{z}, \hat{w}) - \frac{K-1}{2}\log n - \frac{L-1}{2}\log d - \frac{KL\nu}{2}\log nd  , \end{array} $$

where *ν* denotes number of specific parameters for each block while $\ell _{c}(\hat {\Theta }, \hat {\mathbf {z}}, \hat {\mathbf {w}})$ is defined as in Eq. 4 with Θ, **z** and **w** being replaced by their estimates. The model associated with the highest value of the ICL is then selected.

Even if the use of this criterion is a well-established practice in co-clustering applications, Keribin et al. (2015) noted that its consistency has not been proved yet to estimate the number of blocks of a LBM. Additionally, Nagin (2009) and Corneli and Erosheva (2020) point out a bias of the ICL towards overestimation of the number of clusters in the longitudinal context. The validity of the ICL could be additionally undermined by the presence of random effects. As noted by Delattre et al. (2014), standard information criteria have unclear definitions in a mixed effect model framework, since the definition of the actual sample size is not trivial. Given that, common asymptotic approximations are not valid anymore. Even if a proper exploration of the problem from a co-clustering perspective is still missing, we believe that the mentioned issues might also have an impact on the derivation of the criterion in Eq. 7. The development of valid model selection tools for LBM when random effects are involved is out of scope of this work, therefore, operationally, we consider the ICL. Nonetheless, the analyses in Section 4 have to be interpreted with full awareness of the limitations described above.

Additionally note that, to practically evaluate Eq. 7, the *complete-data log-likelihood* is required. As outlined in the previous section, marginalization procedures are needed to compute the marginal densities involved in Eq. 4. As a consequence, the first term Eq. 7 is approximated, thus possibly depending on the considered marginalization scheme. Nonetheless, different approximation strategies have been proposed and their accuracy have been thoroughly tested (see, e.g. Pinheiro and Bates, 1995), showing that the choice of a specific procedure is not strongly influential.

Lastly, since the ICL would serve to the selection of both the number of row and column clusters and the random effect configuration, note that the involved computational time might be rather demanding, also depending on the sample size, the data dimension and the number of observed time occasions. In such situations, resorting to some greedy search strategy, where not all models under evaluations have to be estimated, could be helpful. See, for instance, Keribin et al. (2017) and Corneli et al. (2020).

### Remarks

The model introduced so far inherits the advantages of both its building ingredients, namely the LBM and the SIM. The local independence assumption allows for multivariate data with high-dimensional complex data structures to be handled in a relatively parsimonious way. On the other hand, the characteristics of the model introduce some relevant advantages, in terms of interpretability of the time evolutions of the variables, even in low dimensional settings. The random effects capture differences among the subjects, while curve summaries can be expressed as a function of the mean shape curve. Additionally, resorting to a smoother when modelling the mean shape function, allows for a flexible handling of functional data whereas the presence of random effects make the model effective also in a longitudinal setting. In fact, the borrowing strength mechanism induced by the random effects can handle sparsely and irregularly sampled longitudinal data (James and Sugar, 2003). Finally, we pursue clustering directly on the observed curves, without resorting to intermediate transformation steps, as is done in Bouveyron et al. (2018) where clustering is performed on an intermediate space, spanned by the basis expansion coefficients used to transform the original data, thus possibly endangering the interpretation in terms of the evolution in time. The model, despite its attractive features, introduces some difficulties that require caution, as in the following discussed.

- *Initialization* The M-SEM algorithm encloses different numerical steps which require the suitable specification of starting values. First, the convergence of EM-type algorithms towards a global maximum is not guaranteed; as a consequence they are known to be sensitive to initialization with a good set of starting values being crucial to avoid local solutions. Assuming *K* and *L* to be known, the M-SEM algorithm requires starting values for **z** and **w** in order to implement the first M step. A standard strategy resorts to multiple random initializations: the row and column partitions are sampled independently from multinomial distributions with uniform weights and the one eventually leading to the highest value of the *complete-data log-likelihood* is retained. An alternative approach, possibly accelerating the convergence, is given by a k-means initialization, where two k-means algorithms are independently run for the rows and the columns of $\mathcal {X}$ and the M-SEM algorithm is initialized with the obtained partitions. It has been pointed out (see, e.g. Govaert and Nadif 2013) that the SEM-Gibbs, being a stochastic algorithm, can attenuate in practice the impact of the initialization on the resulting estimates. Finally, note that a further initialization is required, to estimate the nonlinear mean shape function within the M step.
- *Convergence and other numerical problems*. Although the benefits of including random effects in the considered framework are undeniable, parameters estimation is known not to be straightforward in mixed effect models, especially in the nonlinear setting (Harring and Liu, 2016). As noted above, the nonlinear dependence of the conditional mean of the response on the random effects requires multidimensional integration to derive the marginal distribution of the data. While several methods have been proposed to compute the integral, convergence issues are often encountered. In such situations, some strategies can be employed to help with convergence of the estimation algorithm. Examples are to try different sets of starting values, to scale the data prior to the modelling step, or to simplify the structure of the model (e.g. by reducing the number of knots of the B-splines). Addressing these issues often results in considerably higher computational times even when convergence is eventually achieved. Depending on the specific data at hand, it is also possible to consider alternative mean shape formulations, such as polynomial functions, which result in easier estimation procedures. Lastly, note that, if available, prior knowledge about the time evolution of the observed phenomenon may be incorporated in the models to introduce some constraints possibly simplifying the estimation process (see, e.g. Telesca et al., 2012).
- *Identifiability*. The proposed model might inherit some of the identifiability issues of its building blocks, i.e. the latent block model and the shape invariant model. The first one shares the same issues of a standard mixture model. As noted by Keribin et al. (2015), LBM is not identifiable due its invariance to blocks relabelling; this might be a problem when Bayesian estimation procedures are adopted but it is less of an issue when, as in this paper, maximum likelihood estimation is considered. A further source of possible identifiability problems arises in the SIM, as discussed by Lindstrom (1995) and, for a more general but related class of models, by Kneip and Gasser (1988). In this work, to limit the potential issues, we optimize *α*_*i*,2_ on the log-scale by replacing it with $\text {e}^{\alpha _{i,2}}$ in Eq. 1, thus forcing the scale parameters to be positive. This might alleviate the identifiability problems possibly induced by the specific characteristics of the shape function *m*(⋅), such as its closeness under multiplication by -1, which implies that *m*(⋅) = −*m*(⋅) (see Lindstrom, 1995 for further details).
- *Curse of flexibility*. Including random effects for both phase and amplitude shifts and scale transformations might allow for a variety of curves that fit the data well. This flexibility, albeit desirable, sometimes leads to excessive extents, possibly leading to issues with parameter estimation. This is especially true in a clustering framework, where data are expected to exhibit a remarkable heterogeneity. From a practical point of view, our experience suggests that the estimation of the parameters *α*_*ij*,2_ turns out to be the most troublesome, sometimes leading to convergence issues and instability in the resulting estimates.

## Numerical Experiments

### Synthetic Data

This section examines the main features of the proposed approach on some synthetic data. The aim of the simulation study is twofold. The first goal of the analyses consists in exploring the capability of the proposed method to properly partition the data into blocks, also in comparison with some competitors such as the one proposed by Bouveyron et al. (2018) (funLBM in the following) and a double k-means approach, where row and column partitions are obtained separately and subsequently merged to produce blocks. With this regard, we evaluate the results by means of the co-clustering adjusted Rand index (CARI, Robert et al., 2021). This criterion generalizes the adjusted Rand index (Hubert and Arabie, 1985) to the co-clustering framework, and takes the value 1 when the blocks partitions perfectly agree up to a permutation. In order to have a fair comparison with the double *k-means* approach, for which selecting the number of blocks is not straightforward, and to separate the uncertainty due to model selection from the one due to cluster detection, we compared models by considering the number of blocks as known and equal to (*K*_true_,*L*_true_). Consistently, we estimate our model only for the true random effects configuration, being the one considered to generate the data.

As for the second aim of the simulations, we evaluate the performances of the ICL in the developed framework to select both the number of blocks (*K*,*L*) and the random effects configuration.

All the analyses have been conducted in the R environment (R Core Team, 2019) with the aid of nlme package (Pinheiro et al., 2019) to estimate the parameters in the M step, and the splines package to handle the B-spline involved in the common shape function. The code implementing the proposed procedure is available upon request.

**Fig. 4:**
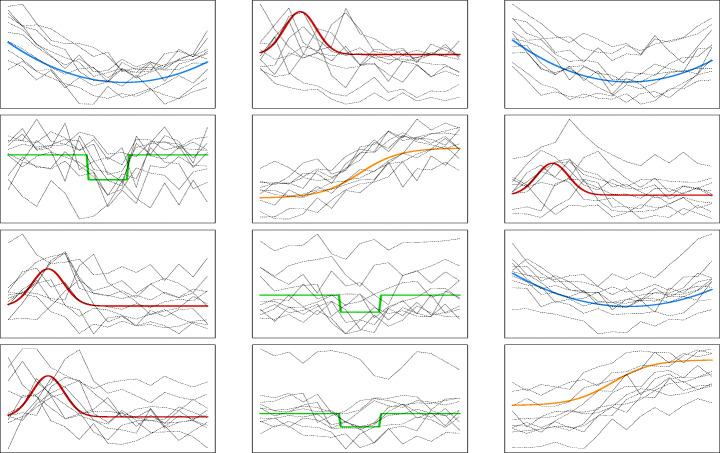
Subsample of simulated curves (black dashed lines) with over-imposed block specific mean shape curves (colored continue lines) employed in the numerical study

The main simulation setup is defined as follows. We generated *B* = 100 Monte Carlo samples of curves according to the general specification in Eq. 3, with block-specific mean shape function *m*_*kl*_(⋅) and both the parameters involved in the error term and the ones describing the random effects distribution being constant across the blocks. In fact, in the light of the considerations made in Section 3.5, the random scale parameter is switched off in the data generative mechanism, i.e. *α*_*ij*,2_ is constrained to be degenerate in zero. We fixed the number of row and column clusters to *K*_true_ = 4 and *L*_true_ = 3. The mean shape functions *m*_*kl*_(⋅) are chosen among four different curves, namely *m*_11_ = *m*_13_ = *m*_33_ = *m*_1_, *m*_12_ = *m*_32_ = *m*_31_ = *m*_41_ = *m*_2_, *m*_21_ = *m*_32_ = *m*_42_ = *m*_3_ and *m*_22_ = *m*_43_ = *m*_4_, as illustrated in Fig. 4 with different color lines, and specified as follows:

We set the other involved parameters to *σ*_*𝜖*,*kl*_ = 0.3, $\mu _{kl}^{\alpha } = (0,0,0)$ and ${\Sigma }_{kl}^{\alpha } = \text {diag}(1,0,0.1)$ $\forall k=1,\dots ,K_{\text {true}}, l=1,\dots ,L_{\text {true}}$. Three different scenarios are considered with generated curves consisting of *T* = 15 equi-spaced observations ranging in [0,1]. As a first baseline scenario, we set the number of rows to *n* = 100 and the number of columns to *d* = 20. The other scenarios are considered in order to obtain insights and indications on the performances of the proposed method when dealing with larger matrices. Coherently in the second scenario, *n* = 500 and *d* = 20 while in the third one, *n* = 100 and *d* = 50 thus increasing respectively the number of samples and features.

Results are reported in Table 1. The proposed method claims excellent performances in all the considered settings, with results notably featured by a very limited variability and sensitivity to changes in *n* or *d*. No clear-cut indications arise from the comparison with funLBM in the baseline scenario, but the latter method shows a larger sensitivity to an increase of data size and dimension, where its performances get worse. The use of an approach which is not specifically conceived for co-clustering, like the the double *k-means*, leads to a stronger degradation of the quality of the partitions. However, not considering jointly the variables and the observations, *k-means* behaves better with increasing dimensions.

**Table 1:** Mean (and std error) of the CARI computed over the simulated samples in the three scenarios. Partitions are obtained using the proposed approach (tdLBM), funLBM and a double k-means approach

	*n* = 100,*d* = 20	*n* = 100,*d* = 50	*n* = 500,*d* = 20
CARI_tdLBM_	0.972 (0.044)	0.988 (0.051)	0.981 (0.020)
CARI_funLBM_	0.950 (0.099)	0.847 (0.183)	0.865 (0.177)
CARI_kmeans_	0.761 (0.158)	0.842 (0.182)	0.809 (0.169)

As for the performances of the ICL, Table 2 shows the fractions of samples where the criterion has led to the selection of each of the considered configurations of (*K*,*L*), with $K,L = 2,\dots ,5$, for models estimated with the proposed method and with funLBM. In all the considered settings, the actual number of co-clusters is the most frequently selected by the ICL criterion, yet a non-negligible tendency to favor overparameterized models, especially for larger sample size, is witnessed, consistently with the comments in Corneli et al. (2020). Conversely, when considering funLBM, the ICL selects the pair (*K*_true_,*L*_true_) in the very large majority of the Monte Carlo simulations.

**Table 2:**
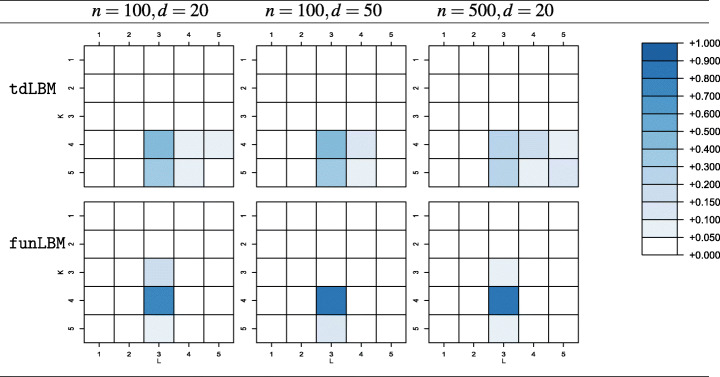
Rate of selection of (*K*,*L*) configurations for the different simulation setups when (*K*_true_ = 4,*L*_true_ = 3)

In addition, the simulations described above have been run on a slightly different setup, where
 While the column partition remains unchanged with respect to the previous setting, in the row partition curves in cluster 3 and 4 differ with respect to either a time shift or a vertical shift only, hence the configuration gets consistent with *K*_true_ = 3 and a TFT layout. The reduced heterogeneity among curves in the new setting simplify the co-cluster detection for both the models, so that results in terms of CARI (not reported for brevity) are almost perfect when they are forced to partition data in the actual number of blocks. However, when the ICL is used to select (*K*,*L*), the different notion of group targeted by funLBM and the proposed model is strongly influencing: on one hand, for our proposal, an overall good behaviour is confirmed when the ICL is used to detect the number of blocks; on the other hand, the same does not apply to funLBM, whose likelihood does not support the designed cluster notion, and the ICL systematically does not select the actual cluster configuration (Table 3).

**Table 3:**
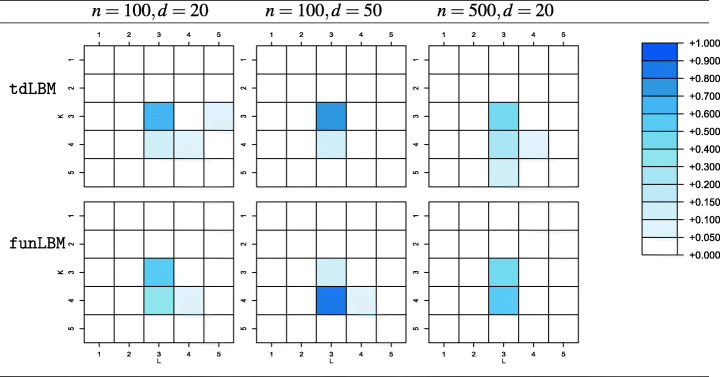
Rate of selection of (*K*,*L*) configurations for the different simulation setups when (*K*_true_ = 3,*L*_true_ = 3)

With respect to the exploration of the performances of the ICL when used to select the random effect configuration (Table 4), we may draw similar considerations to the selection of the number of co-clusters. Here, the ICL selects the true configuration for the majority of the samples in two scenarios while, in the third one, the true model is selected approximately one out of two samples. Nonetheless, also in this case, a tendency to overestimation is visible, with the TTT configuration frequently selected in all the scenarios. In general, the penalization term in Eq. 7 seems to be too weak and overall not completely able to account for the presence of random effects. These results, along with the remarks at the end of Section 3.3, provide a suggestion about a possibly fruitful research direction to provide some suitable adjustments.

**Table 4:** Rate of selection for each random effects configuration in the considered scenarios. Bold cells represents the true data generative model (TFT), blank ones represent percentages equal to zero

		FFF	TFF	FTF	FFT	TTF	TFT	FTT	TTT
	*n* = 100,*d* = 20					1%	**58%**		41%
% of selection	*n* = 100,*d* = 50		2%			1%	**62%**		35%
	*n* = 500,*d* = 20		1%			5%	**47%**		47%

In fact, it is worth noting that when the selection of the number of clusters is the aim, the observed behavior is preferable with respect to underestimation since it does not undermine the homogeneity within a block; this has been confirmed by further analyses suggesting that the additional groups are usually small and arising because of the presence of outliers. As for the random effect configuration, we believe that since the choice impacts the notion of cluster one aims to identify, it should be driven by subject-matter knowledge rather than by automatic criteria. Additionally, the reported analyses are exploratory in nature, aiming to provide general insights on the characteristics of the proposed approach. To limit computational time required to run the large number of models involved in Tables 2, 3, 4, we did not use multiple initializations and we have pre-selected the number of knots for the block-specific mean functions. In practice, we recommend using multiple starting values and carrying out sensitivity analyses on the number of knots to ensure that the conclusions are not affected.

### Applications to Real World Problems

#### French Pollen Data

The data we consider in this section are provided by the *Réseau National de Surveillance Aérobiologique* (RNSA), the French institute which analyzes the biological particles content of the air and studies their impact on human health. RNSA collects data on concentration of pollens and moulds in the air, along with some clinical data, in more than 70 municipalities in France.

The analyzed dataset contains daily observations of the concentration of 21 pollens for 71 cities in France in 2016. Concentration is measured as the number of pollens detected over a cubic meter of air and carried on by means of some pollen traps located in central urban positions over the roof of buildings, in order to be representative of the trend air quality.

The aim of the analysis is to identify homogeneous trends in the pollen concentration over the year and across different geographic areas. For this reason, we focus on finding groups of pollens differentiating one from the others for either the period of maximum exhibition or the time span they are present. Consistently with this choice, we estimate only models with the *y*-axis shift parameter *α*_*ij*,1_ (i.e. *α*_*ij*,2_ and *α*_*ij*,3_ are switched off), for varying number of row and column clusters, and we select the best one via ICL. We consider monthly data by averaging the observed daily concentrations over each month. The resulting dataset may be represented as a matrix with *n* = 71 rows (cities), *p* = 21 columns (pollens) where each entry is a sequence of *T* = 12 time-indexed measurements. Moreover, to practically apply our proposed procedure, we carried out a preprocessing step as we standardized and log-transformed the data, in order to improve the stability of the estimation procedure.

**Fig. 5:**
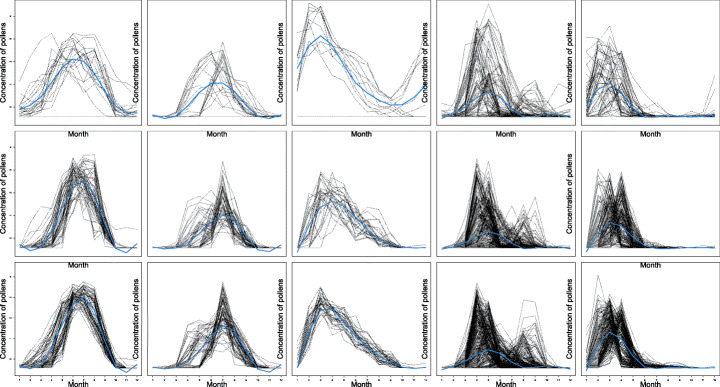
French pollen data results. Curves belonging to each single block with superimposed the corresponding block specific mean curve (in light blue)

Results are graphically displayed in Fig. 5. The ICL selects a model with *K* = 3 row clusters and *L* = 5 column ones. A first visual inspection of the time evolutions reveals that the procedure is able to discriminate the pollens according to their seasonality. Pollens in the first two column groups are mainly present during the summer, with a difference in the intensity of the concentration. In the remaining three groups, pollens are more active during winter and spring months but with a different time persistence and evolution. Column clusters are roughly grouping together trees pollens, distinguishing them from weeds and grass (right panel of Table 5). Results align with the standard four seasons, with groups of pollens from trees mostly present in winter and spring while the ones from grass spreading in the air mainly during the summer months. With respect to the row partition, displayed in the left panel of Table 5, three clusters have been detected, with one roughly corresponding to the Mediterranean region (in blue). The situation, as it concerns the other two clusters, appears to be more heterogeneous. One of these groups (in red) tends to gather cities in the northern region and on the Atlantic coast, mostly featured by oceanic climate, while the other (in green) mainly covers the central part of the country, including Paris and its surrounding area, where climate gradually move to continental characteristics. Digging deeper substantially in the cluster configuration obtained is beyond the scope of this work and may benefit from insights from experts of botanical and geographical disciplines since other factors, as for example the type of environment, with areas being more rural than others, can be strongly influencing.

**Table 5:** French map with superimposed the points indicating the cities colored according to their row cluster memberships (left) and pollens organized by the column cluster memberships (right)

Row groups (cities)	Column groups (pollens)	
	1	Gramineae, Urticaceae
	2	Chestnut, Plantain
	3	Cypress
	4	Ragweed, Mugwort, Birch, Beech,
		Morus, Olive, Platanus, Oak, Sorrel
		Linden
	5	Alder, Hornbeam, Hazel, Ash,
		Poplar, Willow

#### COVID-19 Evolution Across Countries

At the time of writing this paper, an outbreak of infection with severe acute respiratory syndrome coronavirus 2 (SARS-CoV-2) has severely harmed the whole world. Countries all over the world have undertaken measures to reduce the spread of the virus: quarantine and social distancing practices have been implemented, collective events have been canceled or postponed, business and educational activities have been either interrupted or moved online.

While the outbreak has led to a global social and economic disruption, its spreading and evolution, also in relation to the aforementioned non pharmaceutical interventions, have not been the same all over the world (see Flaxman et al., 2020; Brauner et al., 2021 for an account of this in the first months of the pandemic). With this regard, the goal of the analysis is to evaluate differences and similarities among the countries and for different aspects of the pandemic.

Since the overall situation is still evolving, and given that testing strategies have significantly changed across waves, we refer to the first wave of infection, considering the data from the 1st of March to the 4th of July 2020, in order to guarantee the consistency of the disease metrics used in the co-clustering. Moreover we restrict the analysis to the European countries. Data have been collected by the Oxford COVID-19 Government Response Tracker (OxCGRT, Hale et al., 2020) and originally refer to daily observations of the number of confirmed cases and deaths for COVID-19 in each country. We also select two indicators tracking the individual country intervention in response to the pandemic: the Stringency index and the Government response index. Both indicators are recorded on a 0–100 ordinal scale that represents the level of strictness of the policy and accounts for containment and closure policies. The latter indicator also reflects health system policies such as information campaigns, testing strategies and contact tracing.

Data have been pre-processed as follows: daily values have been converted into weekly averages in order to reduce the impact of short term fluctuations and the number of time observations. Rates per 1000 inhabitants have been evaluated from the number of confirmed cases and deaths, and the logarithms applied to reduce the data skewness. All the variables have been standardized.

The resulting dataset is a matrix with *n* = 38 rows (countries), *d*= 4 columns (variables describing the pandemic evolution and containment), observed over a period of *T* = 18 weeks. Unlike the French pollen data, here there is no strong reason to favour one random effect configuration over the others. Conversely, different configurations of random effects would entail different ideas of similarity of virus evolution. Thus, while the presence of random effects would lead to a clustering of similar trends associated to different intensities, speed of evolution and time of onset, switching the random effects off could result in enhancing such differences via the separation of the trends.

**Fig. 6:**
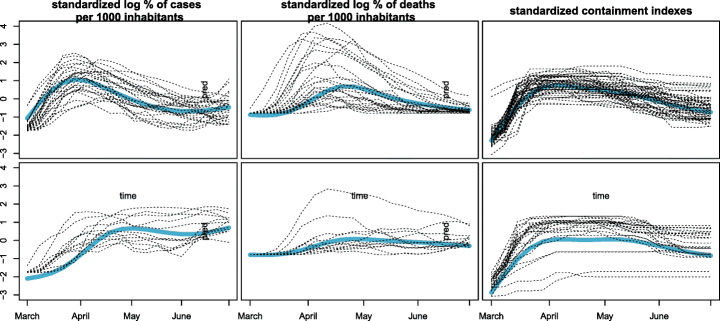
COVID-19 outgrowth results of the best model, with *K* = 2, *L* = 3 and the three random effects on. Curves belonging to each single block with superimposed the associated block specific mean curve (in light blue)

Models have been run for *K* = 1,…,6 row clusters and *L* = 1,2,3 column clusters, and all the 8 possible configurations of random effects. The behaviour of the resulting ICL values supports the remark in Section 4.1, as the criterion favours highly parameterized models. This holds particularly true with regard to the random effects configuration where the larger the number of random effects switched on, the higher the corresponding ICL. Thus, models with all the random effects switched on stand out among the others, with a preference for *K* = 2 and *L* = 3 whose results are displayed in Fig. 6. The obtained partition is easily interpretable: in the column partition, reported on the right panel of Table 6, the containment indexes are grouped together into the same cluster whereas the log-rate of positiveness and death are singleton clusters. Consistently with the random effect configuration, row clusters exhibit a different evolution in terms of cases, deaths and undertaken containment measures: one cluster (in orange in the left panel in Table 6) gathers countries where the virus has spread earlier and caused more losses; here, more severe control measures have been adopted, whose effect is likely seen in a general decreasing of cases and deaths after achieving a peak. The second row cluster (in blue in the map) collects countries for which the death toll of the pandemic seems to be more contained. The virus outbreak generally shows a delayed onset and a slower growth, which does not show a steep decline after reaching the peak, although the containment policies remain high for a long period. Notably, the row partition is also geographical, with the countries with higher mortality all belonging to the Western Europe.

**Table 6:**
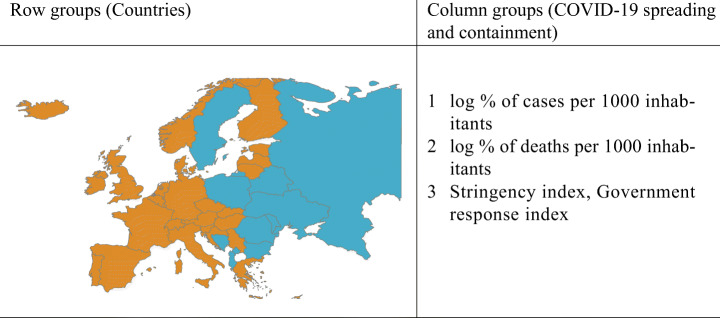
Europe map with countries colored according to their row cluster memberships (left) and variables organized by the column cluster membership (right) for the best ICL model

**Fig. 7:**
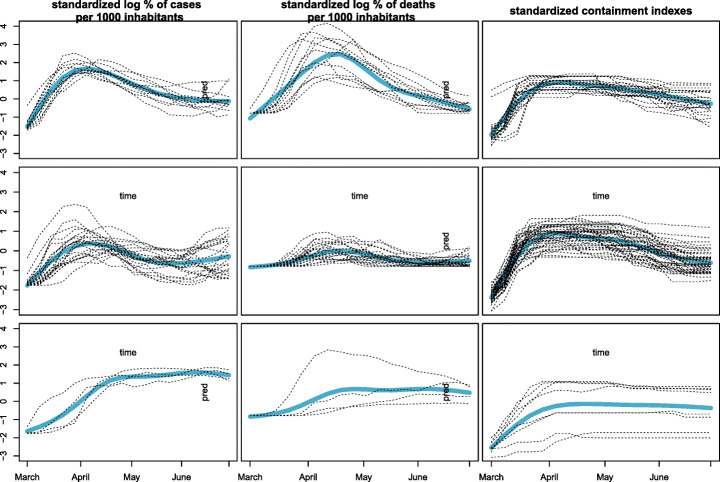
COVID-19 outgrowth results of the model with *K* = 3, *L* = 3 and the three random effects off

To properly show the benefits of considering different random effects configurations in terms of notion and interpretation of the clusters, we also illustrate the partition produced by another model estimated having the three random effects switched off (Fig. 7). Here, we consider *K* = *L* = 3: the column partition remains unchanged with respect to the best model, and the row partition still separates countries by the severity of the impact, yet with the third additional cluster having intermediate characteristics. According to this model, two row clusters feature countries with a similar right-skewed bell-shaped trend of cases and similar policies of containment, yet with a notable difference in the virus lethality. Indeed, the effect of switching *α*_2_ off is clearly noted in the log-rate of death fitting, with two mean curves having similar shapes but different scales. The additional intermediate cluster, less impacted in terms of death rate, is populated by countries from the central-east Europe. The apparent smaller impact of the first wave of the pandemic on the eastern European countries could be explained by several factors ranging from demographic characteristic and more timely closure policies to a different international mobility pattern. Additionally, other factors such as the general economic and health conditions might have prevented accurate testing and tracking policies, so that the actual spreading of the pandemic might have been underestimated.

## Conclusions

Modelling multivariate time-dependent data requires accounting for heterogeneity among subjects, capturing similarities and differences among variables, as well as correlations between repeated measures. In this work, we tackled these challenges by proposing a new parametric co-clustering methodology, recasting the widely known latent block model (Govaert and Nadif, 2013) in a time-dependent fashion. The co-clustering model, by simultaneously searching for row and column clusters, partitions three-way matrices in blocks of homogeneous curves. This approach takes into account the mentioned features of the data while building parsimonious and meaningful summaries. As a data generative mechanism for a single curve, we have considered the *shape invariant model* that has turned out to be particularly flexible when embedded in a co-clustering context. The model allows to describe arbitrary time-evolution patterns while adequately capturing dependencies among repeated measures over time. The proposed method compares favorably with the few existing competitors, producing co-partitions with similar quality as measured by objective criteria, while enjoying some relevant advantages in terms of interpretability and applicability to both functional and longitudinal data. The option of “switching off” some of the random effects, although in principle simplifying the model structure, increases its flexibility, as it allows to encompass different notions of cluster possibly depending on the specific applications and on subject-matter considerations.

While further analyses are required to increase our understanding about the general performance of the proposed model, its application to both simulated and real data has provided good results and highlighted some aspects which are worth further investigation. One interesting direction for future research is studying possible alternatives to the ICL to be used in model selection when the model specification in the LBM framework involves random effects. In addition, alternative choices, for example, for specifying the block mean curves, could be considered and compared with the choices adopted here. Finally, a further direction for future work would be exploring a fully Bayesian approach. This may allow for the incorporation of prior knowledge, when available, within the model and it can lessen the impact of the model selection step, by embedding it automatically within the estimation procedure.
